# 2/1 and 1/1 cubic approximants in the ternary *R*-Cd-Mg (*R* = Y, Er) systems

**DOI:** 10.1107/S2052520621006715

**Published:** 2021-07-25

**Authors:** Tsunetomo Yamada, Nobuhisa Fujita, Farid Labib

**Affiliations:** a Faculty of Science, Department of Applied Physics, Tokyo University of Science, Tokyo 125-8585, Japan; b Institute of Multidisciplinary Research for Advanced Materials, Tohoku University, Sendai 980-8577, Japan

**Keywords:** quasicrystals, approximants, structure analysis, X-ray diffraction

## Abstract

The atomic structures of three cubic approximants (cAPs), Y_12.7_Cd_61.8_Mg_25.5_ [2/1 cAP with space group *Pa* 3, *a* = 25.0654 (1) Å] and Er_12.5_Cd_72.0_Mg_15.5_ [2/1 cAP with *Pa* 3, *a* = 24.9658 (1) Å], and Y_14.5_Cd_66.1_Mg_19.4_ [1/1 cAP with *Im* 3, *a* = 15.4577 (1) Å], were investigated using X-ray structure analysis. The two 2/1  cAPs were found to be isostructural to the binary 2/1 cAP, YbCd_5.8_, which is composed of two building units, namely Tsai-type rhombic triacontahedron (RTH) cluster and double-Friauf polyhedron (DFP). The 1/1 cAP, in contrast, was found to be isostructural to the binary 1/1 cAP, YbCd_6_, which is composed solely of the RTH cluster. The refined structure models of the three cAPs exhibit substitutional disorders due to the Mg content that enters into Cd sites of the parent binary cAPs. In addition, in the 2/1 cAPs, the Y3(Er3) site on the third icosahedron shell of the RTH cluster and two positions inside DFP, Y5(Er5) sites, are partially mixed with Mg. Moreover, characteristic positional disorders owing to orientational disorder in the first shell of the RTH cluster is observed in all the three cAPs. A close relationship between the occupational disorder of the Y3(Er3) and Y5(Er5) sites in 2/1 cAPs and the elemental distribution in their first coordination shells is revealed.

## Abstract 2

The atomic structures of 2/1 and 1/1 quasicrystalline approximants in the ternary *R*-Cd-Mg (*R* = Y, Er) system are revealed using X-ray structural analysis.

## 1. Introduction   

Icosahedral quasicrystals (iQCs) possess long-range order which generates sharp peaks in the diffraction diagram along with a rotational symmetry incompatible with translational symmetry (Shechtman *et al.*, 1984 ▸; Levine & Steinhardt, 1984 ▸). The atomic structure of iQCs can be described within the so-called higher-dimensional crystallography (Janssen *et al.*, 2007 ▸; de Boissieu *et al.*, 2007 ▸), in which the atomic arrangement is generated as a three-dimensional (3D) section of a six-dimensional (6D) hypothetical crystal structure (or *hypercrystal*). The atomic structure can be modified by applying a shear strain to the 6D hypercrystal along the perpendicular space. Such a shear strain is called a *linear phason strain* [see, for instance, Quiquandon *et al.* (1999 ▸) and references therein] and can make real space intersect the 6D hypercrystal at more than one translationally equivalent points simultaneously. The real-space section would then be periodic at least in one direction. A series of cubic crystal structures with varying periodicity can be generated by introducing isotropic linear phason strains, and these are hereafter referred to as cubic approximants (cAPs). Each cAP is attached to a rational number, *q*/*p*, which belongs to the sequence, 1/0, 1/1, 2/1, …, *F*_*n*_/*F*_*n*−1_, … that converges to the golden mean, τ = [1 + (5)^1/2^]/2. Here, *F*_*n*_ is the *n*th Fibonacci number. The relation between the 6D unit-cell parameter *a*_6D_ of the hypercrystal and the 3D unit-cell parameter *a*_*q*/*p*_ of a *q*/*p* cAP is expressed as (Goldman & Kelton, 1993 ▸) 

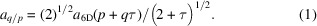




**Figure 1:**
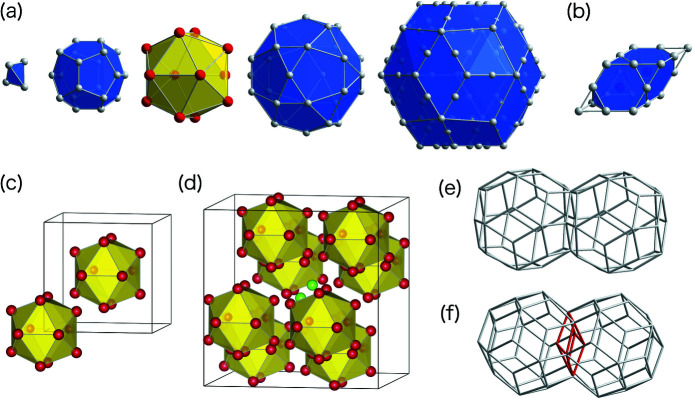
Building blocks of i-YbCd_5.7_ and related cAPs. The red and grey spheres represent Yb and Cd atoms, respectively. (*a*) The shell structure of the Tsai-type cluster (RTH unit). (*b*) The atomic decoration of the DFP unit. (*c*,*d*) The arrangements of Tsai-type clusters in the 1/1 (YbCd_6_) and 2/1 (YbCd_5.8_) cAPs, respectively, depicted using the third icosahedral shells. (*e*,*f*) Pairs of Tsai-type clusters connected by *b* and *c* linkages, respectively, depicted using the outermost polyhedra.

In 2000, the first stable iQCs as binary alloys were discovered in the Yb-Cd and Ca-Cd systems (Tsai *et al.*, 2000 ▸; Guo *et al.*, 2000 ▸). Seven years later, Takakura *et al.* performed a structure analysis of the binary iQC, i-YbCd_5.7_, using single-crystal X-ray diffraction (SXRD), leading to the first ever accurate structure determination of an iQC (Takakura *et al.*, 2007 ▸). This was made possible due largely to the high structural quality without chemical disorder, which could not be hoped for with any ternary iQC. The reported atomic structure involves two main building blocks, *i.e.* rhombic triacontahedron (RTH) and acute rhombohedron units as depicted in Fig. 1 ▸. The RTH unit, also known as the Tsai-type cluster, consists of five constituent shells (ordered from the centre): Cd_4_ tetrahedron, Cd_20_ dodecahedron, Yb_12_ icosahedron, Cd_30_ icosidodecahedron and Cd_92_ RTH. The acute rhombohedron unit contains two Yb atoms on its longer body diagonal, while the vertices and mid-edge positions are occupied by Cd atoms. The latter can also be interpreted as double Friauf polyhedron (DFP), as depicted in Fig. 1 ▸(*b*). In these binary alloy systems, both 1/1 and 2/1 cAPs form and their chemical compositions are very close to those of the iQCs, *i.e.* YbCd_6_ and CaCd_6_ for 1/1 cAPs and YbCd_5.8_ and Ca_13_Cd_76_ for 2/1 cAPs (Pay Gómez & Lidin, 2001 ▸, 2003 ▸; Takakura *et al.*, 2001 ▸). The atomic structure of the 1/1 cAPs is described as a body-centred cubic packing of RTHs, while that of the 2/1 cAP involves both RTHs and DFPs, as depicted in Figs. 1 ▸(*c*) and 1 ▸(*d*), respectively.

In the atomic structure of the iQC, 2/1 and 1/1 cAPs, RTHs are connected to each other by two kinds of linkages, *i.e. b* and *c* linkages (Takakura *et al.*, 2007 ▸). A *b* linkage connects two RTHs along a twofold axis such that a rhombic face is shared between the two, as shown in Fig. 1 ▸(*e*). A *c* linkage connects two RTHs along a threefold axis such that the two are weakly interpenetrated with each other (sharing an obtuse rhombohedron), as shown in Fig. 1 ▸(*f*). The whole arrangement of RTHs thus defines a network connected by these linkages, wherein the local configuration of each RTH is characterized by the numbers, *N*_*b*_ and *N*_*c*_, of *b* and *c* linkages, respectively, connecting the RTH to its adjacent neighbours. In the refined structure model of the YbCd_5.7_ iQC, the twelvefold sphere-packing model with icosahedral symmetry is employed as the underlying *bc* network (Henley, 1986 ▸). This model has 18 distinct local configurations, in which the most frequent ones are characterized by (*N*_*b*_, *N*_*c*_) = (7, 5) and (6, 6) (Takakura, 2008 ▸; Takakura & Strzałka, 2017 ▸). By contrast, in the 2/1 or 1/1 cAPs, every RTH has a unique local configuration with the corresponding numbers being (6, 7) or (6, 8), respectively. Note that neither of the latter two is a member of the 18 allowed local configurations in the structure model of the iQC.

Recently, new binary iQCs were discovered in the *R*-Cd (*R* = Gd to Tm, and Y) and Sc-Zn systems (Canfield *et al.*, 2010 ▸; Goldman *et al.*, 2013 ▸). Soon afterwards, structure analysis was performed on some of these binary iQCs including i-GdCd_7.88_, i-DyCd_7.50_, i-TmCd_7.28_ and i-ScZn_7.33_, where they were found to be isostructural to i-YbCd_5.7_ (Yamada *et al.*, 2016*a* ▸,*b* ▸). Yet, an important difference lies in the presence of occupational disorder at two kinds of rare-earth sites, *i.e.* the third icosahedron shell of RTH and diagonal positions of DFP. Because of the occupational disorder, the compositional weights of rare-earth elements are significantly reduced from that of i-YbCd_5.7_, in which both of these sites are purely occupied by Yb. In fact, the intricate nature of the occupational disorder has not been fully clarified yet as the previous refinements by Yamada *et al.* used a single global parameter to determine the mixing ratio of the two species at each of the icosahedron shell sites. In contrast, the occupational disorder at any of the rare-earth sites most likely depends on the local atomic environment which derives from the arrangement of neighbouring clusters. A way to accommodate the intrinsic effect of local atomic environment in a refinement would be to introduce multiple occupation probability parameters for different sub-classes of rare-earth sites.

2/1 cAPs are often highlighted as the simplest cAPs that involve both the RTH and DFP units. This implies that a suitable 2/1 cAP could provide insight into the occupational disorder that persists in the binary iQCs, i-*R*-Cd (with *R* ≠ Yb) and i-Sc-Zn. To date, however, the only reported 2/1 cAPs in binary alloy systems, YbCd_5.8_ and Ca_13_Cd_76_, are free from chemical disorder, in that the icosahedron shell and diagonal positions of DFP are purely occupied by Yb and Ca, respectively (Pay Gómez & Lidin, 2001 ▸). Meanwhile, new 2/1 cAPs were found in ternary *R*-Cd-Mg (*R* = Gd to Tm, and Y) alloy systems (Labib *et al.*, 2019 ▸, 2020 ▸) with the concentration of the large atoms (*i.e. R*) being significantly lower than that in YbCd_5.8_ and Ca_13_Cd_76_ 2/1 cAPs. This strongly suggests the presence of occupational disorder at the third icosahedron shell of RTH and the two positions inside DFP. The main purpose of this study is to analyze the atomic structure of these ternary 2/1 cAPs, hoping to gain insight into the nature of the occupational disorder. The acquired information could possibly be applied to refining the 6D structure model of related iQCs including those in the binary *R*-Cd and Sc-Zn systems.

In this paper, the atomic structure of ternary Y-Cd-Mg 2/1 and 1/1 cAPs and the Er-Cd-Mg 2/1 cAP is investigated in detail using X-ray single-crystal structure analysis. Our refined structure models show that the Cd sites of the parent binary Yb-Cd cAPs are partially substituted with Mg. In addition, we find that in both the 2/1 cAPs, Mg also partially substitutes some *R* sites which belong to a unique crystallographic position in the third icosahedron shell of the RTH unit and to the diagonal positions of the DFP unit. As for the 1/1 cAP, the icosahedral shell was found to be fully occupied by Y. The mixing ratios of *R*/Mg and Cd/Mg in the refined structures are found to be strongly correlated with the local chemical environment of individual sites.

The paper is organized as follows: in §2 we described the experimental detail, and in §3 we present the results of SXRD. In §4 we present the result of structure refinements of the Y-Cd-Mg 2/1 and 1/1 cAPs and the Er-Cd-Mg 2/1 cAP. In §5 we describe the refined structures. In the last section, we summarized the important characteristics of the atomic structures.

## 2. Experimental   

In this study, single grains of the Y-Cd-Mg 2/1 and 1/1 cAPs and the Er-Cd-Mg 2/1 cAP that had been previously synthesized by the self-flux method were studied by SXRD experiment. The details of synthesis and characterization can be found elsewhere (Labib *et al.*, 2019 ▸; Labib *et al.*, 2020 ▸). The evaluated compositions of these alloys using scanning electron microscopy with energy-dispersive X-ray spectroscopy are Y_12.7_Cd_61.8_Mg_25.5_ and Er_12.5_Cd_72.0_Mg_15.5_ for the 2/1 cAPs and Y_14.5_Cd_66.1_Mg_19.4_ for the 1/1 cAP. SXRD experiments for the Y-Cd-Mg and Er-Cd-Mg 2/1 cAPs were carried out with an XtaLAB synergy single-crystal diffractometer equipped with Hybrid Pixel Array Detector (HyPix6000, Rigaku) using Mo *K*α radiation (λ = 0.71073 Å), at ambient temperature. The data collection was performed with a crystal-to-camera distance of 35 mm. An SXRD experiment for the Y-Cd-Mg 1/1 cAP was carried out with a single-crystal diffractometer equipped with a CCD detector (Saturn 724HG, Rigaku) using Mo *K*α radiation at 100 K. The data collection was performed with a crystal-to-camera distance of 45 mm. In each of these experiments, a single-crystal specimen of about 50 µm in diameter was selected from crushed fragments of the alloy and mounted on a MiTeGen MicroMount^TM^. The indexing of Bragg reflections, integration of diffraction intensities and absorption correction were performed utilizing the *CrysAlisPRO* software (Rigaku Oxford Diffraction, 2018 ▸).

## 3. Structural analysis   

### 3.1. Single-crystal X-ray diffraction   

For the Y-Cd-Mg 1/1 cAP, 64 031 reflections were observed with a resolution limit of 0.51 Å. All the reflections were indexed using a body-centred cubic lattice with a unit-cell parameter of 15.4577 (1) Å. For the Y-Cd-Mg 2/1 cAP, 439 634 reflections were observed with a resolution limit of 0.8 Å. All the reflections were indexed using a primitive cubic lattice with a unit-cell parameter of 25.0654 (1) Å. The unit-cell parameters are close to those of the 1/1 cAC (15.48 Å) and 2/1 cAC (25.05 Å), respectively, estimated from a 6D unit-cell parameter of the YCd_7.48_ iQC (7.955 Å) using equation (1) (Goldman *et al.*, 2013 ▸). The Laue class 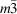 was assumed while performing an empirical absorption correction using spherical harmonics, implemented in SCALE3 ABSPACK module in *CrysAlisPRO*. The space groups 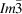 and 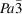 were suggested by the space group determination module, GRAL, in *CrysAlisPRO* for the 1/1 and 2/1 cAPs, which resulted in 2579 and 5382 unique reflections with internal agreement factors (*R*_int_) being 0.0292 and 0.038, respectively. Reciprocal space sections perpendicular to twofold, threefold and pseudo-fivefold directions as reconstructed from the SXRD data sets are provided in the supporting information, in which no superstructure reflection can be spotted meaning that all the observed reflections satisfy the reflection conditions of the space groups.

For the Er-Cd-Mg 2/1 cAP, 443 875 reflections were observed with a resolution limit of 0.8 Å. All the reflections were indexed using a primitive cubic lattice with a unit-cell parameter of 24.9658 (1) Å. The same process as above resulted in 5307 unique reflections with *R*_int_ being 0.036 with the space group 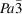 .

### 3.2. Phase retrieval   

*Ab initio* phasing was carried out on the diffraction data using the charge-flipping algorithm (Oszlányi & Sütő, 2004 ▸, 2005 ▸) with the *SUPERFLIP* program (Palatinus & Chapuis, 2007 ▸). Preliminary structural models were subsequently constructed from the resulting electron densities by utilizing the *JANA2006* program (Petříček *et al.*, 2014 ▸).

For the Y-Cd-Mg 1/1 cAP, the preliminary model was isostructural to the *R*Cd_6_ 1/1 cAP (Pay Gómez & Lidin, 2001 ▸), with an RTH unit comprising the second dodecahedron (M2, M4), third icosahedron (Y1), fourth icosidodecahedron (M3, M5), and fifth RTH (M1, M3, M4, M6) shells. The first shell was absent at this stage. Besides, the model seemed unreliable in terms of elemental distribution because wrong assignment of elements to the atomic sites was highly possible due to the presence of both occupational and positional disorders. Therefore, instead of accepting the preliminary model as it is, a hypothetical model of the binary YCd_6_ 1/1 cAP was built by elemental replacements, such that Y and Cd atoms are arranged at Y1 and M1–M6 positions, respectively, to be used as the initial model for the refinement. As described in the next subsection, occupational and positional disorders were taken into account by introducing chemical mixings and site splittings while iterating refinement cycles that used the difference Fourier synthesis method.

For the Y-Cd-Mg 2/1 cAP, the preliminary model was isostructural to the Ca_13_Cd_76_ 2/1 cAP that involves both the RTH and DFP units (Pay Gómez & Lidin, 2001 ▸). A difference between the latter two structures was evident in the first shell: we found a triangle as the first shell in the ternary model while it is a tetrahedron in the Ca_13_Cd_76_ 2/1 cAP. Similar to the case of the 1/1 cAP, we built a hypothetical model of the binary Y-Cd 2/1 cAP to be used as the initial model. Here, Y atoms were arranged in the third icosahedron shell (Y1–Y4) and at the diagonal positions of DFP (Y5), corresponding to the Ca sites in the Ca_13_Cd_76_ 2/1 cAP. Cd atoms on the other hand were arranged at the other sites (M1–M27), which include the first triangle (M27), second dodecahedron (M8, M9, M13, M14, M15, M16, M19, M21), fourth icosidodecahedron (M6, M7, M10, M11, M12, M17, M18, M20, M22, M25) and fifth RTH (M1, M2, M3, M4, M5, M6, M7, M8, M10, M11, M12, M15, M16, M17, M18, M23, M24, M26) shells. An initial model for the Er-Cd-Mg 2/1 cAP was built in the same way as described above.

### 3.3. Structure analysis of 1/1 cAP   

For the Y-Cd-Mg 1/1 cAP, structure parameters including atomic coordinates, site occupation factors (SOFs) and atomic displacement parameters (ADPs) were refined by utilizing *SHELXL* program (Sheldrick, 2015 ▸) against 2562 unique reflections. After iterating structure-refinement cycles utilizing the difference Fourier synthesis method, the first shell (M7, 48*h*) was introduced with an SOF being 1/6, which amounts to a total of four atoms in each cluster centre. In addition, mixtures of Cd/Mg were assigned to M1–M7 and the mixing ratios were refined, which is followed by introducing site splits. After iterating refinement cycles, we found that each of the M2–M5 sites splits into two, and that the M7 site splits into three. ADPs of Cd and Mg occupying the same position were restrained to be identical for the splitting sites of M2–M5, while a common ADP was set for the triad of M7. The fitting converged with reliability indexes of *R*1 = 0.0224, *wR*2 = 0.0509 and *S* = 1.245. Here, the weight, *w*, was estimated using equation: 

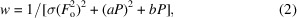

where *a* = 0.0144 and *b* = 31.4293 are fitting parameters and 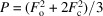 . The refined composition (Y_14.5_Cd_66.1_Mg_19.4_) was in good agreement with the experimental one (Y_14.18_Cd_64.95_Mg_20.86_). Basic crystallographic information and parameters for the data collection and structure refinement are summarized in Table 1 ▸. These data are also provided in the relevant crystallographic information file (CIF) provided in supporting information.

**Figure 2:**
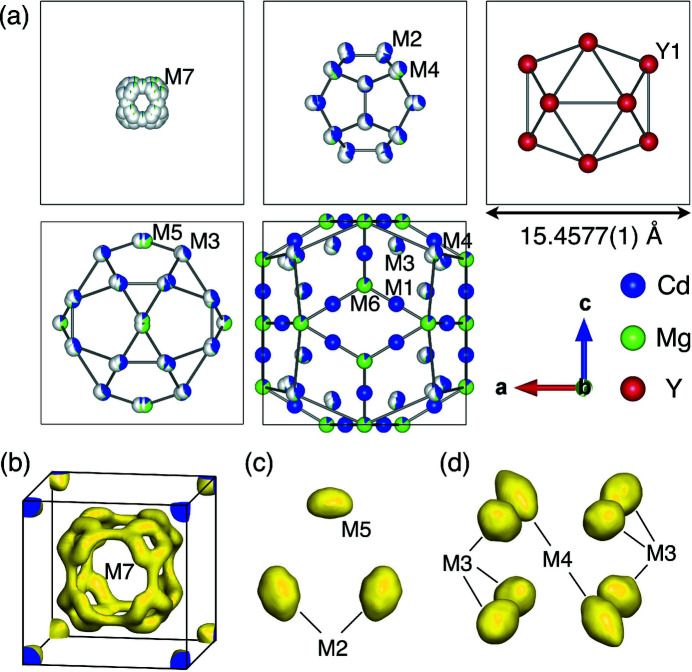
Representation of the atomic coordinates (as spheres), the occupancies (as colored fractions of spheres, with white meaning unoccupied) and the calculated electron-density isosurfaces in the refined structure of the Y-Cd-Mg 1/1 cAP. (*a*) shell structures in the RTH units. Electron-density isosurfaces at (*b*) M7, (*c*) M2 and M5, (*d*) M3 and M4 as obtained from the maximum entropy method. The electron-density isosurfaces were drawn at the 5 e^−^Å^−3^ level.

Fig. 2 ▸(*a*) presents the constituent shells of the RTH unit in the refined structure. Figs. 2 ▸(*b*)–2 ▸(*d*) present electron-density isosurfaces showing characteristic atomic displacements at M2, M3, M4, M5 and M7. These isosurfaces were generated by *Dysnomia* (Momma *et al.*, 2013 ▸), a computer program to perform the maximum entropy method, with *R* = 0.013 and *wR* = 0.015. M7 (first shell) is split into M7a, M7b and M7c, each of which is occupied by Cd/Mg with a mixing ratio of 0.72/0.28. The second dodecahedron shell consists of two independent atomic positions, M2 and M4, which are occupied by Cd/Mg with mixing ratios of 0.95/0.05 and 0.80/0.20, respectively. We found the former splits into M2a and M2b, separated by 0.415 (6) Å and the latter splits into M4a and M4b, separated by 0.399 (1) Å. The corresponding electron density isosurfaces for M2 and M4 are somewhat elongated as shown in Figs. 2 ▸(*c*) and 2 ▸(*d*), respectively. The third icosahedron shell consists of Y1 which is fully occupied by Y. The fourth icosidodecahedron shell consists of two independent positions, M3 and M5, for which the corresponding electron density isosurfaces are somewhat elongated as shown in Figs. 2 ▸(*d*) and 2 ▸(*c*), respectively. Two split sites were introduced for both M3 [M3a and M3b, separated by 0.309 (7) Å] and M5 [M5a and M5b, separated by 0.406 (3) Å]. Both M3 and M5 are occupied by Cd/Mg with mixing ratios of 0.90/0.10 and 0.28/0.72, respectively. The fifth RTH shell consists of four independent positions, M1, M3, M4 and M6. M1 comes with a single site that is fully occupied by Cd, whereas M6 comes with a single site that is occupied by Cd/Mg with a mixing ratio of 0.13/0.87. M3 and M4 are shared with the fourth and second shells, respectively.

When comparing the resulting structure of the 1/1 cAP to that of Yb_13.3_Cd_70.3_Mg_16.5_ 1/1 cAP (Yamada *et al.*, 2017 ▸), we found the following differences. First, the overall shape of the electron density isosurfaces for the first shell of the Y-Cd-Mg 1/1 cAP [Fig. 2 ▸(*b*)] differs from that of the Yb_13.3_Cd_70.3_Mg_16.5_ 1/1 cAP. Second, the Yb_13.3_Cd_70.3_Mg_16.5_ 1/1 cAP exhibits a much larger M4-splitting of 1.10 (2) Å than that in the Y-Cd-Mg 1/1 cAP [namely, 0.399 (1) Å]. Third, the Yb_13.3_Cd_70.3_Mg_16.5_ 1/1 cAP entails an extra site, M8, with a fractional occupancy at the centre of each cube spanned by M3 and M4 (Yamada *et al.*, 2017 ▸), whereas there is no need for such an extra site for the Y-Cd-Mg 1/1 cAP.

These differences are worth having a closer look. In the Yb_13.3_Cd_70.3_Mg_16.5_ 1/1 cAP, the electron density of the first shell is concentrated at the corners of a cube oriented towards threefold directions parallel to 〈111〉 from the cluster centre. Only four atoms can enter these positions to form a tetrahedron in one of the two possible orientations related through 90° rotation. The disorder associated with the tetrahedral orientation is called the ‘type-I disorder’ by Pay Gómez & Lidin (2003 ▸), wherein each of the cube corners are semi-occupied. On the other hand, in the Y-Cd-Mg 1/1 cAP, the electron density shows a ring of maxma around the threefold centre as seen in Fig. 2 ▸(*b*). This is interpreted as a result of site splitting that takes place at every corner of a tetrahedron. The associated disorder, called the ‘type-II disorder’ by Pay Gómez & Lidin (2003 ▸), indeed coexists with the type-I disorder in the Y-Cd-Mg 1/1 cAP because the site splitting is observed at every corner of the cube in the electron density map. The noted difference in the magnitude of M4 splitting can be connected to the different types of disorder in the first shell. Note that the unrealistic short distance [1.75 (2) Å] between M7 in the first shell and M4 in the second shell in the Yb_13.3_Cd_70.3_Mg_16.5_ 1/1 cAP prohibits adjacent M4 and M7 sites to be occupied simultaneously, thus causing a large split distance of M4 to avoid collision with an occupied M7 site. More specifically, the large splitting of M4 results from a push or pull by the first shell along a threefold axis (Yamada *et al.*, 2017 ▸). Note that this can provide a large enough space to accept an extra atom at M8, which is located at the midpoint of each c linkage, when two opposing atoms at M4 are pulled away towards cluster centres. On the other hand, in the case of the Y-Cd-Mg 1/1 cAP, the four atoms in the first shell are deviated from the *c* linkages, so that the push or pull mechanism is suppressed. Hence, there would not be enough space to accept an extra atom at M8 even when the two opposing atoms at M4 were pulled away. Of course, this can be taken the other way around. The cube spanned by M3 and M4 is slightly smaller and hence possibly stiffer in the Y-Cd-Mg 1/1 cAP than that in the Yb_13.3_Cd_70.3_Mg_16.5_ 1/1 cAP due to the smaller unit cell. This would hinder the push or pull mechanism to distort the cube and, in compensation, the tetrahedral vertices would split in the first shell. In both the scenarios, the contrast between the two structures in terms of the occupational and positional disorders at M4 and M8 can be closely connected with the different types of orientational disorder in the first shell represented by M7.

### 3.4. Structure analysis of 2/1 cAP   

For the Y-Cd-Mg 2/1 cAP, structure refinement was performed using 5382 unique reflections. Mixtures of Cd and Mg were assigned to atomic sites, M1–M27, and their SOFs along with the coordinates and ADPs were refined. Besides, a mixing ratio of Cd/Mg on the first shell (M27) was fixed to be 0.3/0.1, based on the refinement result of the Y-Cd-Mg 1/1 cAP. After iterating structure-refinement cycles utilizing the difference Fourier synthesis method, a partial atomic occupation at the centre of the RTH unit was revealed by residual electron densities at around (0.1545 0.1545 0.1545) (8*c*). Since previous authors had shown that the cluster centres in various Tsai-type 1/1 cAPs could be partially or fully occupied by a single large (*i.e.* rare-earth) atom instead of the first tetrahedron shell (Fornasini *et al.*, 2008 ▸; Lin & Corbett, 2010 ▸; Deguchi *et al.*, 2015 ▸; Gebresenbut *et al.*, 2016 ▸, 2020 ▸; Yamada *et al.*, 2017 ▸), we arranged an atomic site for Y atom at the centre position and refined the SOF assuming that the innermost part of the RTH unit is either a single Y (labelled Y6) atom or the first triangle shell. The subsequent refinement cycles converged with reliability indexes of *R*1 = 0.0601, *wR*2 = 0.1703 and *S* = 1.097. Here, the parameters in the weight function of equation (2) were determined to be *a* = 0.0289 and *b* = 339.1513. The resulting structure, however, exhibits large residual electron densities with a maximum of 5.47 e^−^ Å^−3^ and a minimum of −5.48 e^−^ Å^−3^. The minimum, in particular, is located at a distance of 0.45 Å from the Y3 site, implying that Y3 has a chemical mixing with the lighter element (*i.e.* Mg) rather than with the heavier Cd.

In an improved model, mixtures of Y/Mg were assigned to all the Y sites (Y1–Y5), and their SOFs were refined accordingly. The refinement converged with reliability indexes of *R*1 = 0.0439, *wR*2 = 0.1129 and *S* = 1.158. The resulting mixing ratios of Y/Mg were 0.50/0.50 and 0.74/0.26 for Y3 and Y5, respectively, while the other Y sites all within the third icosahedron shell were found to be fully occupied by Y atoms. At this stage, however, the resulting structure still exhibits high residual densities with a maximum of 3.66 e^−^ Å^−3^, which led to Alert level A in checkCIF (Spek, 2020 ▸). Prominent residual peaks are located nearby the atomic sites in the second dodecahedron shell. As a further improvement, we introduced five additional sites, M28–M32, with fractional occupancies of Cd. Here, their ADPs were fixed to be 0.01 Å^2^ and the possible mixing of Cd with Mg was not used on account of the relatively small gain compared to the associated instabilities in refinement calculations. The fitting converged with reliability indexes of *R*1 = 0.0380, *wR*2 = 0.0907 and *S* = 1.166. Here, the fitting parameters in the weight function *w* of equation (2) were determined to be *a* = 0.0289 and *b* = 339.1513. The refined composition (Y_12.71_Cd_60.47_Mg_26.82_) was in good agreement with the experimental one (Y_12.7_Cd_61.8_Mg_25.5_). Basic crystallographic information and parameters for the data collection and structure refinement are summarized in Table 1 ▸. The same data can also be found in the relevant CIF provided in supporting information.

**Figure 3:**
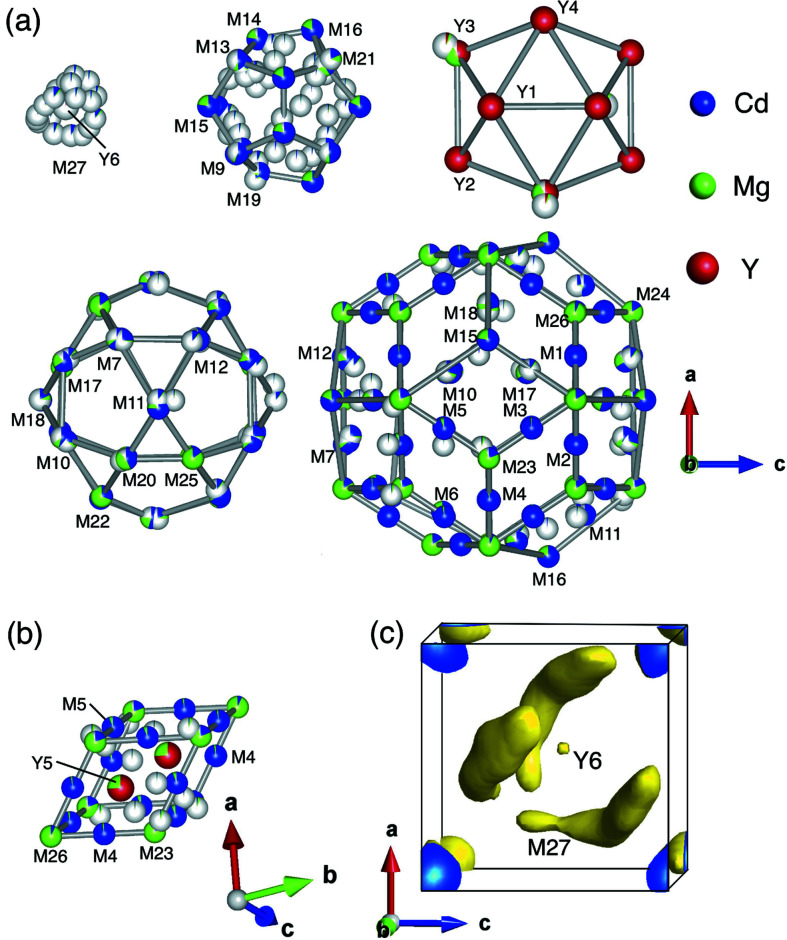
Representation of the atomic coordinates (as spheres), the occupancies (as colored fractions of spheres, with white meaning unoccupied) and the calculated electron-density isosurfaces in the refined structure of the Y-Cd-Mg 2/1 cAP. (*a*) shell structures in the RTH unit. (*b*) structure of the DFP unit. (*c*) electron-density isosurfaces at Y6 and M27 as obtained from the maximum entropy method. The electron-density isosurfaces were drawn at the 5 e^−^Å^−3^ level.

Figs. 3 ▸(*a*) and 3 ▸(*b*) present the constituent shells of the RTH unit and atomic arrangement within the DFP unit, respectively, in the resulting structure of the 2/1 cAP. Fig. 3 ▸(*c*) presents electron-density isosurfaces in the central part of RTH generated by *Dysnomia* (Momma *et al.*, 2013 ▸) with *R* = 0.024 and *wR* = 0.019. The innermost shell of the RTH unit in Fig. 3 ▸(*a*) is a superposition of two mutually exclusive entities. On one hand, the single site Y6 with SOF being 0.067 models the weak electron density peak presented in the centre of Fig. 3 ▸(*c*). On the other hand, seven split sites (M27a–M27g), which are occupied by Cd/Mg with the fixed mixing ratio of 0.3/0.1, are used to model the three arc shaped isosurfaces surrounding the centre. The resulting SOFs for M27a–M27g correspond to about 2.4 atoms present at M27 in the refined structure. Note that the number of atoms at M27 may differ depending on the mixing ratio of Cd/Mg which was fixed to 0.3/0.1 in present analysis. The second dodecahedron shell consists of eight independent positions which are preferentially occupied by Cd. In particular, three of the positions on the *c* linkages are represented by sites labelled M8, M15 and M16 and are fully occupied by Cd/Mg without site splitting. [Note that the M8 and M21 sites in the second shell are on the threefold symmetry axis, while the former is hidden behind M9 in Fig. 3 ▸(*a*).] These sites are on average at a distance of 4.40 (6) Å from the cluster centre, while the other sites that show site splitting are on average at a distance of 3.94 (7) Å, indicating that the second dodecahedron shell is distorted along *c* linkages. The distortion can be interpreted as a result of a push or pull by the first shell, in the same manner as the first tetrahedron shell results in large site-splitting in the 1/1 cAP. The third icosahedron shell consists of four independent positions (Y1–Y4), all of which except Y3 are non-mixed and represented by fully occupied Y sites. The Y3 position, on the other hand, is occupied by a mixture of Y/Mg, while it also shows site splitting which is represented by two split sites labelled Y3a and Y3b lying 0.73 (2) Å apart. The fourth icosidodecahedron shell consists of ten independent positions and is found to be distorted in such a way that the triangle faces intersecting with c linkages becomes wider. In addition, sites on the *b* linkages (M22, M25) are found to be preferentially occupied by Mg without site splitting. In contrast, the other positions in the fourth shell show site splitting and these are preferentially occupied by Cd or Cd/Mg. The fifth RTH shell consists of 17 independent positions. The vertex positions of RTH are preferentially occupied by Mg except for M15 and M16 which are shared with neighbouring second dodecahedron shells. On the other hand, the edge-centre sites are preferentially occupied by Cd. Since all the six rhombic faces of the DFP unit are shared with adjacent RTH shells, the vertex and edge-centre sites of the DFP are preferentially occupied by Mg and Cd, respectively. The diagonal positions (Y5) of the DFP unit are found to be occupied by a mixture of Y/Mg with a ratio of 0.74/0.26.

For the Er-Cd-Mg 2/1 cAP, the structure refinement converged with reliability indexes of *R*1 = 0.0560, *wR*2 = 0.1335 and *S* = 1.209. Here, large residual electron densities were observed with a maximum of 4.10 e^−^ Å^−3^ inside the second dodecahedron shell, which led to Alert level A in checkCIF. Whereas the structure refinement is still in progress, at the current stage, the refined structure exhibits partial mixings of Mg in the Er3 site belonging to the third icosahedron shell of the RTH unit and in the diagonal positions (Er5) of the DFP unit. These observations suggest that the crystal structure is similar to that of the Y-Cd-Mg 2/1 cAP.

### 3.5. Occupational disorder in 1/1 and 2/1 cAPs   

In a recent study, one of the present authors analyzed the site preferences of Mg atoms in ternary Yb-Cd-Mg iQC and 1/1 cAP. He showed that the atomic sites that are preferentially occupied by Mg have fewer Yb atoms in the first coordination shell (Yamada *et al.*, 2017 ▸). Below, the refined structures of the Y-Cd-Mg 2/1 and 1/1 cAPs are examined from a similar point of view.

The coordination number, *CN*, the number of Y sites in the first coordination shell, *CN*(Y), the total site occupancies of individual elements in the first coordination shell, *N*(Y), *N*(Cd) and *N*(Mg), and the mixing ratios of Y/Cd/Mg for each atomic site in the Y-Cd-Mg 1/1 and 2/1 cAPs are provided in Tables 2 ▸ and 3 ▸, respectively. By definition, there is an equality, *CN* = *N*(Y)+*N*(Cd)+*N*(Mg). Here, *CN* of each of the atomic sites was determined from the Voronoi tessellation of a modified structure in which split sites with the same number label in the refined structure were merged into a single site by averaging according to their SOFs as the weighting coefficients. Note that some sites in the 1/1 AP exhibit non-integer values of *CN* in Table 2 ▸ since these include M7 site with the SOF being 1/6 in their first coordination shell. In addition, the *CN* in Table 3 ▸ was calculated by assuming that the SOF of M27 in the 2/1 AP equals to 1.

As seen in Table 2 ▸, the *CN*(Y)s of the M1–M6 sites in the Y-Cd-Mg 1/1 cAP are either 2 or 3. Note that the M5 and M6 sites whose *CN*(Y) is equal to 2 are preferentially occupied by Mg, while the other sites whose *CN*(Y) is equal to 3 are preferentially occupied by Cd. Such a selective occupation rule is in common with the Yb_13.3_Cd_70.3_Mg_16.5_ 1/1 cAP (Yamada *et al.*, 2017 ▸).

**Figure 4:**
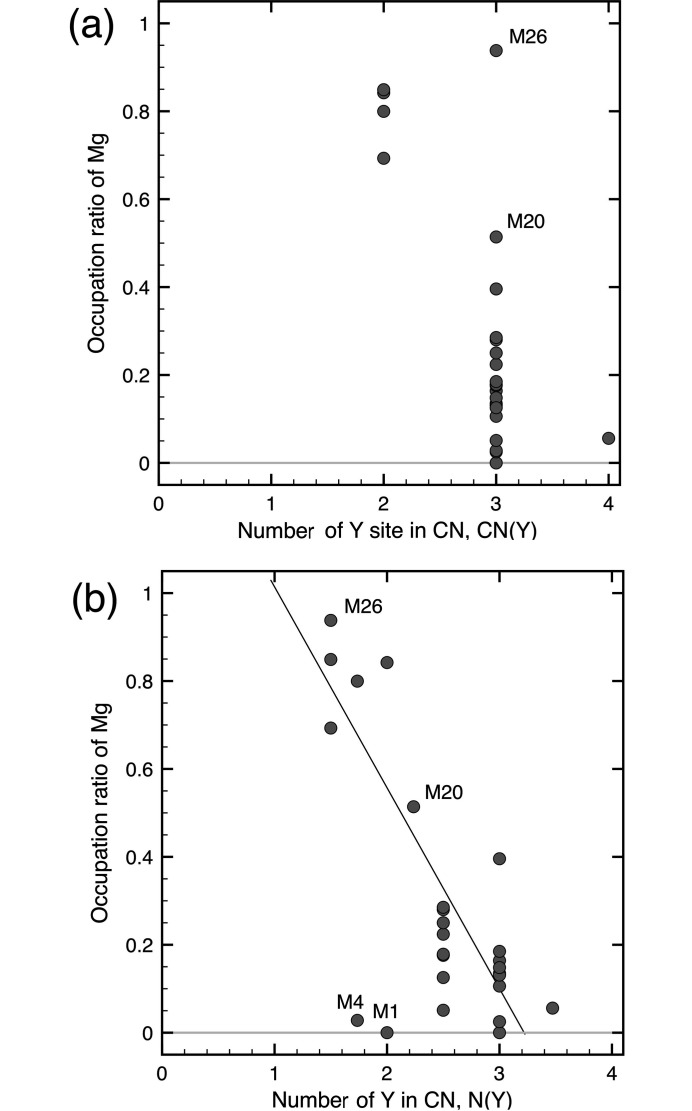
Correlations between Mg occupancy and local environment for M1–M26 in the Y-Cd-Mg 2/1 cAP. The occupation ratio of Mg is plotted against (*a*) *CN*(Y) and (*b*) *N*(Y). A few of the data points are marked by their site labels.

In the Y-Cd-Mg 2/1 cAP, *CN*(Y) and *N*(Y) can take different values because of the mixing of Y and Mg at Y3 and Y6. In the following, we examine how the Mg occupancies at M1–M26 are correlated with the values of *CN*(Y) and *N*(Y). Fig. 4 ▸(*a*) plots the relative Mg occupancies against *CN*(Y) for each of the M1–M26 sites in the Y-Cd-Mg 2/1 cAP. Observe that the *CN*(Y) values range from 2 to 4 and that Mg atoms preferentially occupy the sites where *CN*(Y) is equal to 2. Besides, the M26 site with *CN*(Y) being 3 may appear exceptionally selective for Mg. Yet, since three Y3 positions occupied by mixtures of Y and Mg are included in the first coordination shell of M26, the actual number of Y atoms around M26 is only 1.5 on average. In Fig. 4 ▸(*b*), the relative Mg occupancies are plotted against the average number of Y atoms, *N*(Y), in the first coordination shell for each of the M1–M26 sites in the Y-Cd-Mg 2/1 cAP. With the values of *N*(Y) ranging from 1.50 to 3.47, a decreasing trend in the Mg occupancy as a function of *N*(Y) is evident. It turns out that M26 closely follows this trend and so does M20, whose Mg occupancy equals to 0.51 at *N*(Y) = 2.24 lying in the middle of the data range. Similar observations in the case of the Er-Cd-Mg 2/1 cAP are presented in supporting information. The correlation between the site selectivity of Mg and the local arrangement of *R* atoms demands future explanation based on electronic structure calculations. In fact, the site selectivity of Mg appears to be affected not only by the *R* population but also by the relative population of Cd/Mg in the first coordination shell. The obvious deviation of M1 and M4 from the linear trend in the plot can be attributed to the high Mg population in the first coordination shell. Note that both the latter sites correspond to mid-edge positions of the RTH shell and are preferentially occupied by Cd despite that their *N*(Y) are lower than 2.

**Figure 5:**
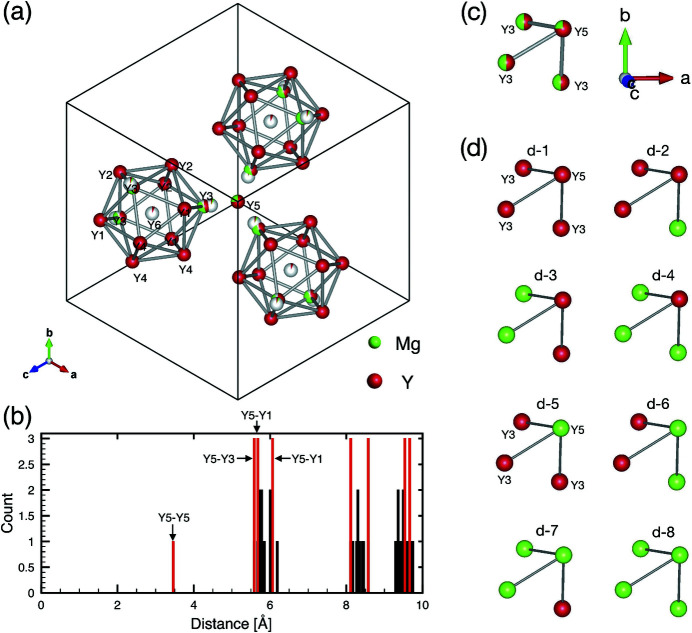
Positions and distribution of atomic distances for Y sites in the refined structure. (*a*) Selected positions of Y1–Y6 in the unit cell projected along a threefold axis parallel to [111]. An icosahedral shell (Y1–Y4) centered at Y6 is drawn together with their associates related by threefold rotations around the [111] axis passing through Y5. (*b*) Distribution of Y–Y atomic distances up to 10 Å. Y–Y pairs which contains (Y5) are emphasized in red. (*c*) Average structure and (*d*) eight different arrangements at Y3 and Y5 sites.

Let us now turn our attention to the local coordination of each of the Y sites (except the fractionally occupied cluster centre, Y6) in the Y-Cd-Mg 2/1 cAP. Fig. 5 ▸(*a*) shows Y sites belonging to the third icosahedron shells (Y1–Y4) of three RTH units as well as the diagonal positions (Y5) of a DFP unit arranged around the [111] axis. The distribution of Y–Y distances up to 10 Å is presented in Fig. 5 ▸(*b*). The *CN* value of each of the Y1–Y5 sites in the 2/1 cAP is either 16 or 17 as shown in Table 3 ▸. Y3 and Y5 are the only mixed sites occupied by Y and Mg. We note that the following observations differentiate these mixed sites from the other pure Y sites. First, the distance between the two diagonal positions of DFP, Y5–Y5, is the shortest (3.46 Å) among the Y–Y distances in the structure. Second, three Y3 positions from different RTH units lie close to a Y5 site, as seen in Fig. 5 ▸(*a*), with the Y3–Y5 distance being 5.59 Å, giving the second shortest Y–Y distance. One can also notice that, apart from the short Y-Y distances, the first coordination shells of these Y sites are much richer in Mg [with *N*(Mg) = 4.62 as listed in Table 3 ▸] than those of the other Y sites.

Fig. 5 ▸(*c*) shows a local configuration of the Y5 site with its nearest three Y3 sites, in which eight symmetrically unique arrangements of Y and Mg atoms are allowed as shown in Fig. 5 ▸(*d*). The average compositions of the two sites are given as a weighted average (with a total weight of unity) of these eight distinct arrangements. However, given that the mixing ratios of Y/Mg are approximately 1/1 and 3/1 at Y3 and Y5, respectively, only three degrees of freedom can be fixed leaving five free parameters for the weighting. As a compromise, one may tentatively assume that the configurations, d-2, d-3 and d-6, in Fig. 5 ▸(*d*) are the only allowed configurations, as they give relatively even mixing between Y and Mg. Then the weights would be determined as 1/4, 1/2 and 1/4 for d-2, d-3 and d-6, respectively. Note that this is only one of the possibilities, while we find no further clue in the diffraction data (*e.g.* in diffuse scatterings) as to the local atomic correlations.

## 4. Conclusion   

The atomic structure of the two 2/1 cAPs in the ternary Y-Cd-Mg and Er-Cd-Mg systems, and the 1/1 cAP in the Y-Cd-Mg system was investigated by X-ray single-crystal analysis. The resulting atomic structures have been discussed with an emphasis on positional and occupational disorders.

The atomic structure of the Y-Cd-Mg 1/1 cAP was found to be isostructural to the parent binary YCd_6_ 1/1 cAP which consists of the RTH unit. The structure analysis revealed that the M5 site on the fourth icosidodecahedron and the M6 site on the fifth RTH shell are preferentially occupied by Mg, while the M1–M4 are preferentially occupied by Cd. This selective Mg occupation is similar to that reported in the Yb_13.3_Cd_70.3_Mg_16.5_ 1/1 cAP. A difference between these two average atomic structures was found in the type of positional disorder in the first tetrahedron shell. The influence of the latter on a positional disorder in the outer part of the RTH unit was explained in terms of the push-pull mechanism.

The atomic structure of the Y-Cd-Mg 2/1 cAP was found to be isostructural to the parent binary YbCd_5.8_ 2/1 cAP which consists of the RTH and DFP units. We found a preferential occupation of Mg at the M22 and M25 sites on the fourth icosidodecahedron shell locating on the *b* linkages and the vertex sites of the fifth RTH shell, with exceptions of the M15 and M16 sites. Furthermore, the resulting structure exhibits a partial Mg substitution in the Y3 site on the third icosahedron shell and one in the Y5 site lying on the long diagonal of DFP. In addition to the above occupational disorder, we found a significant number of split sites in the average structure presenting positional disorder.

Finally, the site selectivity of the constituting elements found in the 1/1 and 2/1 cAPs was analyzed based on the coordination number (*CN*). In particular, we found an important correlation between the Mg occupancy of an individual site and the number of *R* atoms included in the first coordinating shell, which is in common with Yb-Cd-Mg iQC and 1/1 cAP.

## Supplementary Material

Crystal structure: contains datablock(s) YCdMg-1-1-cAP, YCdMg-2-1-cAP. DOI: 10.1107/S2052520621006715/dq5051sup1.cif

Structure factors: contains datablock(s) YCdMg-1-1-cAP. DOI: 10.1107/S2052520621006715/dq5051YCdMg-1-1-cAPsup2.hkl

Structure factors: contains datablock(s) YCdMg-2-1-cAP. DOI: 10.1107/S2052520621006715/dq5051YCdMg-2-1-cAPsup3.hkl

Supporting information file. DOI: 10.1107/S2052520621006715/dq5051sup4.pdf

CCDC references: 2092766, 2092767

## Figures and Tables

**Table 1:** Experimental details

	YCdMg 1/1-cAP	YCdMg 2/1-cAP
Crystal data		
Chemical formula	Y_24_Cd_109.89_Mg_35.29_	Y_90.40_Cd_432.91_Mg_177.41_
*M*_r_	15344.63	61013.94
Crystal system, space group	Cubic, *I* *m*\overline{3}	Cubic, *P* *a*\overline{3}
Temperature (K)	100	298
*a* (Å)	15.4577 (1)	25.0654 (1)
*V* (Å^3^)	3693.47 (3)	15747.9 (1)
*Z*	1	1
*F*(000)	6660	26441
*D* *_x_* (Mg m^−3^)	6.899	6.434
Radiation type	Mo *K*α	Mo *K*α
μ (mm^−1^)	25.01	22.54
Crystal size (mm)	0.08 × 0.08 × 0.07	0.65 × 0.60 × 0.50

Data collection		
Diffractometer	MicroMax007HF - Saturn 724 HG	XtaLAB Synergy R, HyPix
Absorption correction	Multi-scan (*CrysAlis PRO*). Empirical absorption correction using spherical harmonics, implemented in SCALE3 ABSPACK scaling algorithm.	Multi-scan (*CrysAlis PRO*). Empirical absorption correction using spherical harmonics, implemented in SCALE3 ABSPACK scaling algorithm.
*T*_min_, *T*_max_	0.505, 1.000	0.766, 1.000
No. of measured, independent and observed [*I* > 2σ(*I*)] reflections	64 031, 2579, 2562	422 818, 5382, 5128
*R*_int_	0.029	0.037
(sin θ/λ)_max_ (Å^−1^)	0.976	0.625

Refinement		
Refinement composition	Y_14.18_Cd_64.95_Mg_20.86_	Y_12.71_Cd_60.47_Mg_26.82_
*R*[*F* ^2^ > 2σ(*F* ^2^)], *wR*(*F* ^2^), *S*	0.022, 0.051, 1.25	0.038, 0.091, 1.17
No. of reflections	2579	5382
No. of parameters	76	438
No. of restraints	0	6
Δρ_max_, Δρ_min_ (e Å^−3^)	2.37, −1.36	1.84, −1.36

Computer programs: *CrysAlis PRO* 1.171.39.46 (Rigaku Oxford Diffraction, 2018 ▸), *SUPERFLIP* (Palatinus & Chapuis, 2007 ▸), *SHELXL2016/6* (Sheldrick, 2015 ▸).

**Table 2:** Coordinate number (*CN*), number of constituent atoms in the *CN, N*(Y), *N*(Cd) *N*(Mg), atomic occupations in the atomic structures of the Y-Cd-Mg 1/1 cAP Sites indicated by * and † are common to vertices and edge centres of the RTH shell, respectively.

Shell	Site	*CN*	*CN*(Y)	*N*(Y)	*N*(Cd)	*N*(Mg)	Y/Cd/Mg
Dodecahedron	M2	9.33	3	3	4.70	1.64	0/0.95/0.05
	M4^*^	13.17	3	3	9.06	1.10	0/0.79/0.21
Icosahedron	Y1	16.33	0	0	13.31	3.03	1/0/0
Icosidodecahedron	M3^†^	11	3	3	5.79	2.21	0/0.90/0.10
	M6	8	2	2	3.48	2.52	0/0.13/0.87
RTH	M1	12	3	3	5.40	3.60	0/1/0
	M5	9	2	2	4.37	2.63	0/0.28/0.72

**Table 3:** Coordinate number (*CN*), number of Y sites in the *CN*, *CN*(Y), number of constituent atoms in the *CN*, *CN*(Y), *N*(Cd), *N*(Mg) and atomic occupations in the atomic structures of the Y-Cd-Mg 2/1 cAP Sites indicated by * and † are common to vertices and edge centres of the RTH shell, respectively.

Shell	Site	*CN*	*CN*(Y)	*N*(Y)	*N*(Cd)	*N*(Mg)	Y/Cd/Mg
Dodecahedron	M8*	13	3	3.00	8.20	1.80	0/0.87/0.13
	M9	11	3	2.50	4.71	3.80	0/0.95/0.05
	M13	10	3	2.50	4.77	2.73	0/0.88/0.13
	M14	11	3	2.50	5.25	3.25	0/0.82/0.18
	M15*	13	3	2.50	7.84	2.56	0/0.78/0.22
	M16*	14	3	3.00	8.51	2.49	0/0.82/0.19
	M19	11	3	2.50	5.61	2.89	0/0.72/0.29
	M21	12	3	3.00	6.61	2.39	0/0.60/0.40
Icosahedron	Y1	17	0	0	13.36	3.64	1/0/0
	Y2	16	0	0	12.51	3.50	1/0/0
	Y3	16	0	0	11.38	4.62	0.50/0/0.50
	Y4	16	0	0	12.07	3.93	1/0/0
Icosidodecahedron	M6†	13	3	3.00	7.37	2.63	0/0.87/0.14
	M7†	13	3	3.00	7.06	2.94	0/0.89/0.11
	M10†	13	3	3.00	7.19	2.81	0/0.84/0.16
	M11†	12	3	3.00	5.98	3.02	0/0.85/0.15
	M12†	13	3	2.50	7.38	3.13	0/0.82/0.18
	M17†	13	3	2.50	7.32	3.19	0/0.75/0.25
	M18†	13	3	2.50	7.35	3.15	0/0.72/0.28
	M20	15	3	2.24	8.15	4.62	0/0.49/0.51
	M22	15	2	1.50	9.03	4.47	0/0.31/0.69
	M25	15	2	1.50	9.29	4.21	0/0.15/0.85
Edge centre of RTH	M1	12	3	2.00	4.77	5.23	0/1/0
	M2	12	3	3.00	5.09	3.91	0/1/0
	M3	12	3	3.00	5.14	3.86	0/0.98/0.03
	M4	12	3	1.74	4.64	5.63	0/0.97/0.03
	M5	12	4	3.47	4.95	3.58	0/0.94/0.06
Vertices of RTH	M23	13	2	1.74	7.90	3.36	0/0.20/0.80
	M24	12	2	2.00	7.66	2.34	0/0.16/0.84
	M26	12	3	1.50	8.39	2.11	0/0.06/0.94
Two sites inside DFP	Y5	16	1	0.74	10.64	4.62	0.74/0/0.26
