# Supplementary material for: 2/1 and 1/1 cubic approximants in the ternary R-Cd-Mg (R = Y, Er) systems
Source: Acta Crystallogr B Struct Sci Cryst Eng Mater. 2021 Jul 25;77(Pt 4):638–48. doi: 10.1107/S2052520621006715 (PMC8409366; doi:10.1107/S2052520621006715)
Supplement: Supplementary file 4 [file b-77-00638-sup4.pdf]

Acta Cryst B

STRUCTURAL SCIENCE
CRYSTAL ENGINEERING
MATERIALS

Volume 77 (2021)

Supporting information for article:

2/1 and 1/1 cubic approximants in the ternary *R*-Cd-Mg (*R* = Y, Er) systems

Tsunetomo Yamada, Nobuhisa Fujita and Farid Labib

Table S1; Atomic coordinates, site occupation factors (SOFs), equivalent isotropic displacement parameters (U_{eq}) and Wyckoff position (WP) for Y-Cd-Mg 1/1 cAP.

Site	Atom	SOF	x	y	z	U_{eq} (Å^2)	WP
Y1	Y	1	0.19045(2)	0.29796(2)	0	0.00782(4)	24g
M1	Cd	1	0.40568(2)	0.34344(2)	0	0.00780(3)	24g
M2a	Cd/Mg	0.59(1)/0.0327(6)	0.41105(9)	0.5	-0.2671(2)	0.0144(2)	24g
M2b	Cd/Mg	0.36(1)/0.0199(6)	0.5	0.7586(3)	0.5968(1)	0.0114(4)	24g
M3a	Cd/Mg	0.53(2)/0.060(2)	0.3056(2)	0.3895(2)	-0.15487(8)	0.0124(2)	48h
M3b	Cd/Mg	0.37(2)/0.043(2)	0.3384(2)	0.2035(2)	0.1270(4)	0.0155(3)	48h
M4a	Cd/Mg	0.384(1)/0.100(1)	0.33261(5)	0.33261(5)	-0.33261(5)	0.0175(3)	16f
M4b	Cd/Mg	0.409(1)/0.107(1)	0.34752(6)	0.34752(6)	-0.34752(6)	0.0203(3)	16f
M5	Cd/Mg	0.138(2)/0.362(2)	0.48686(7)	0.5	-0.09465(6)	0.0124(3)	24g
M6	Cd/Mg	0.128(3)/0.872(3)	0.31342(7)	0.5	0	0.0173(3)	12e
M7a	Cd/Mg	0.0218(7)/0.057(2)	0.5401(6)	0.5591(6)	-0.4087(5)	0.0235(9)	48h
M7b	Cd/Mg	0.0218(7)/0.0218(7)	0.5436(5)	0.5889(5)	-0.4334(7)	0.0235(9)	48h
M7c	Cd/Mg	0.0178(8)/0.047(2)	0.5	0.5762(8)	-0.4197(8)	0.0235(9)	24g

Table S2; Atomic coordinates, site occupation factors (SOFs), equivalent isotropic displacement parameters (U_{eq}) and Wyckoff position (WP) for Y-Cd-Mg 2/1 cAP.

Site	Atom	SOF	x	y	z	Ueq (Å^2)	WP
Y1	Y	1	0.15274(3)	0.34086(3)	0.03900(3)	0.0101(2)	24d
Y2	Y	1	0.54011(3)	0.34541(3)	0.03197(3)	0.0113(2)	24d
Y3a	Y/Mg	0.453(2)/0.455(2)	0.76835(6)	0.34419(5)	0.03403(7)	0.0159(4)	24d
Y3b	Y/Mg	0.046(2)/0.046(2)	0.5584(8)	0.7159(7)	0.6558(6)	0.0159(4)	24d
Y4	Y	1	0.33959(3)	0.26916(3)	0.15410(3)	0.0145(2)	24d
Y5	Y/Mg	0.74(1)/0.26(1)	0.96014(3)	0.46014(3)	0.03986(3)	0.0103(5)	8c
Y6	Y	0.067(4)	0.3455(4)	0.8455(4)	0.6545(4)	0.005	8c
M1	Cd	1	0.65673(2)	0.75197(2)	0.59671(2)	0.0122(1)	24d
M2	Cd	1	0.65462(2)	0.94072(2)	0.59536(2)	0.0117(1)	24d
M3	Cd/Mg	0.975(4)/0.025(4)	0.53627(2)	0.74904(2)	0.40618(2)	0.0130(2)	24d
M4	Cd/Mg	0.972(4)/0.028(4)	0.59636(2)	0.65787(2)	0.56071(2)	0.0140(2)	24d
M5a	Cd/Mg	0.935(1)/0.05533(8)	0.43973(2)	0.59293(2)	0.46379(2)	0.0140(2)	24d
M5b	Cd/Mg	0.009(1)/0.00055(8)	0.441(2)	0.557(2)	0.440(2)	0.01	24d
M6	Cd/Mg	0.865(4)/0.135(4)	0.55677(3)	0.91375(3)	0.53039(3)	0.0232(3)	24d
M7a	Cd/Mg	0.25(3)/0.030(3)	0.7169(4)	0.8587(4)	0.4227(4)	0.0188(8)	24d
M7b	Cd/Mg	0.64(3)/0.076(3)	0.7136(2)	0.8543(2)	0.4348(4)	0.0188(8)	24d
M8	Cd/Mg	0.869(7)/0.131(7)	0.44822(4)	0.94822(4)	0.55178(4)	0.0301(5)	8c
M9a	Cd/Mg	0.555(3)/0.0303(2)	0.4449(2)	0.7467(2)	0.5559(2)	0.0171(5)	24d
M9b	Cd/Mg	0.345(3)/0.0189(2)	0.4364(3)	0.7565(3)	0.5634(3)	0.0171(5)	24d
M9c	Cd/Mg	0.025(2)/0.00135(8)	0.405(1)	0.773(1)	0.5586(9)	0.0171(5)	24d
M9d	Cd/Mg	0.016(1)/0.00086(8)	0.379(2)	0.785(2)	0.548(2)	0.0171(5)	24d
M9e	Cd/Mg	0.009(1)/0.009(1)	0.405(2)	0.804(2)	0.536(2)	0.01	24d
M10a	Cd/Mg	0.54(2)/0.106(3)	0.4258(3)	0.7138(2)	0.4462(3)	0.0165(8)	24d
M10b	Cd/Mg	0.29(2)/0.058(3)	0.4339(4)	0.7155(4)	0.4571(3)	0.0165(8)	24d
M11a	Cd/Mg	0.750(2)/0.1308(4)	0.59259(4)	0.65493(4)	0.36330(5)	0.0183(3)	24d
M11b	Cd/Mg	0.092(2)/0.0161(4)	0.5923(5)	0.6540(4)	0.3450(4)	0.0183(3)	24d
M11c	Cd/Mg	0.010(1)/0.0017(2)	0.597(2)	0.688(2)	0.346(2)	0.01	24d
M12a	Cd/Mg	0.72(1)/0.154(2)	0.6490(2)	0.7592(1)	0.71624(4)	0.0228(6)	24d
M12b	Cd/Mg	0.11(1)/0.023(2)	0.7723(6)	0.7193(4)	0.6341(4)	0.0228(6)	24d
M13a	Cd/Mg	0.485(3)/0.0698(4)	0.7484(1)	0.7517(2)	0.4447(2)	0.0157(5)	24d
M13b	Cd/Mg	0.320(3)/0.0460(4)	0.7594(3)	0.7448(2)	0.4348(3)	0.0157(5)	24d
M13c	Cd/Mg	0.037(2)/0.0053(3)	0.7724(7)	0.7478(6)	0.4086(9)	0.0157(5)	24d

Site	Atom	SOF	x	y	z	Ueq (Å^2)	WP
M13d	Cd/Mg	0.022(2)/0.0032(3)	0.781(2)	0.743(1)	0.428(2)	0.0157(5)	24d
M13e	Cd/Mg	0.010(1)/0.0015(2)	0.749(2)	0.750(2)	0.366(2)	0.01	24d
M14a	Cd/Mg	0.770(2)/0.1671(4)	0.70124(4)	0.65493(3)	0.40176(4)	0.0220(2)	24d
M14b	Cd/Mg	0.040(2)/0.0087(3)	0.3720(9)	0.7137(6)	0.6508(6)	0.0220(2)	24d
M14c	Cd/Mg	0.012(1)/0.0026(3)	0.345(2)	0.714(2)	0.690(2)	0.01	24d
M15	Cd/Mg	0.776(4)/0.224(4)	0.50779(4)	0.84540(3)	0.71601(3)	0.0288(3)	24d
M16	Cd/Mg	0.815(5)/0.185(5)	0.68190(5)	0.84637(3)	0.78448(3)	0.0356(4)	24d
M17a	Cd/Mg	0.66(3)/0.22(1)	0.7619(2)	0.7867(2)	0.5496(4)	0.0243(8)	24d
M17b	Cd/Mg	0.09(3)/0.03(1)	0.756(1)	0.781(1)	0.539(2)	0.0243(8)	24d
M18a	Cd/Mg	0.54(2)/0.207(8)	0.5984(1)	0.8456(1)	0.6394(4)	0.0181(8)	24d
M18b	Cd/Mg	0.18(2)/0.070(8)	0.6001(4)	0.8445(4)	0.6533(6)	0.0181(8)	24d
M19a	Cd/Mg	0.048(2)/0.0191(7)	0.4653(7)	0.8642(6)	0.5971(6)	0.0261(6)	24d
M19b	Cd/Mg	0.517(2)/0.2066(9)	0.4887(1)	0.8466(1)	0.6008(1)	0.0261(6)	24d
M19c	Cd/Mg	0.150(2)/0.0600(9)	0.5026(5)	0.8460(5)	0.5934(5)	0.0261(6)	24d
M20a	Cd/Mg	0.43(2)/0.45(2)	0.6378(3)	0.5852(2)	0.4726(1)	0.0223(8)	24d
M20b	Cd/Mg	0.06(2)/0.06(2)	0.651(1)	0.574(1)	0.4758(8)	0.0223(8)	24d
M21a	Cd/Mg	0.409(2)/0.268(1)	0.7588(2)	0.7588(2)	0.7588(2)	0.049(2)	8c
M21b	Cd/Mg	0.177(2)/0.116(1)	0.7448(2)	0.7448(2)	0.7448(2)	0.017(2)	8c
M21c	Cd/Mg	0.019(2)/0.012(1)	0.776(1)	0.780(1)	0.757(2)	0.017(2)	24d
M22	Cd/Mg	0.307(4)/0.693(4)	0.55271(6)	0.77449(7)	0.53272(6)	0.0298(6)	24d
M23a	Cd/Mg	0.191(2)/0.762(6)	0.5338(1)	0.65643(9)	0.4703(1)	0.0288(7)	24d
M23b	Cd/Mg	0.009(2)/0.037(6)	0.549(2)	0.641(2)	0.447(2)	0.0288(7)	24d
M24	Cd/Mg	0.158(5)/0.842(5)	0.65617(8)	0.84427(7)	0.53540(8)	0.0209(7)	24d
M25	Cd/Mg	0.151(5)/0.849(5)	0.6431(1)	0.7290(1)	0.47100(9)	0.0358(9)	24d
M26	Cd/Mg	0.062(8)/0.938(8)	0.65793(9)	0.65793(9)	0.65793(9)	0.014(2)	8c
M27a	Cd/Mg	0.115(4)/0.038(1)	0.8638(7)	0.6896(6)	0.4065(5)	0.045(2)	24d
M27b	Cd/Mg	0.175(4)/0.058(1)	0.8426(4)	0.7055(3)	0.3903(4)	0.045(2)	24d
M27c	Cd/Mg	0.089(4)/0.030(1)	0.8263(7)	0.7177(6)	0.3712(6)	0.045(2)	24d
M27d	Cd/Mg	0.056(2)/0.0188(8)	0.4039(6)	0.8786(6)	0.6294(7)	0.045(3)	24d
M27e	Cd/Mg	0.075(5)/0.025(2)	0.3725(5)	0.834(1)	0.5922(6)	0.045(3)	24d
M27f	Cd/Mg	0.029(2)/0.0096(7)	0.780(1)	0.676(1)	0.327(1)	0.045(3)	24d
M27g	Cd/Mg	0.024(2)/0.0081(7)	0.792(2)	0.666(1)	0.296(2)	0.045(3)	24d
M28	Cd/Mg	0.018(2)	0.752(1)	0.636(1)	0.444(1)	0.01	24d

Site	Atom	SOF	x	y	z	Ueq (Å^2)	WP
M29	Cd/Mg	0.0164(2)	0.558(1)	0.865(1)	0.748(1)	0.01	24d
M30	Cd/Mg	0.011(2)	0.622(2)	0.847(2)	0.782(2)	0.01	24d
M31	Cd/Mg	0.012(2)	0.786(2)	0.682(2)	0.463(2)	0.01	24d
M32	Cd/Mg	0.013(2)	0.461(2)	0.716(2)	0.506(2)	0.01	24d

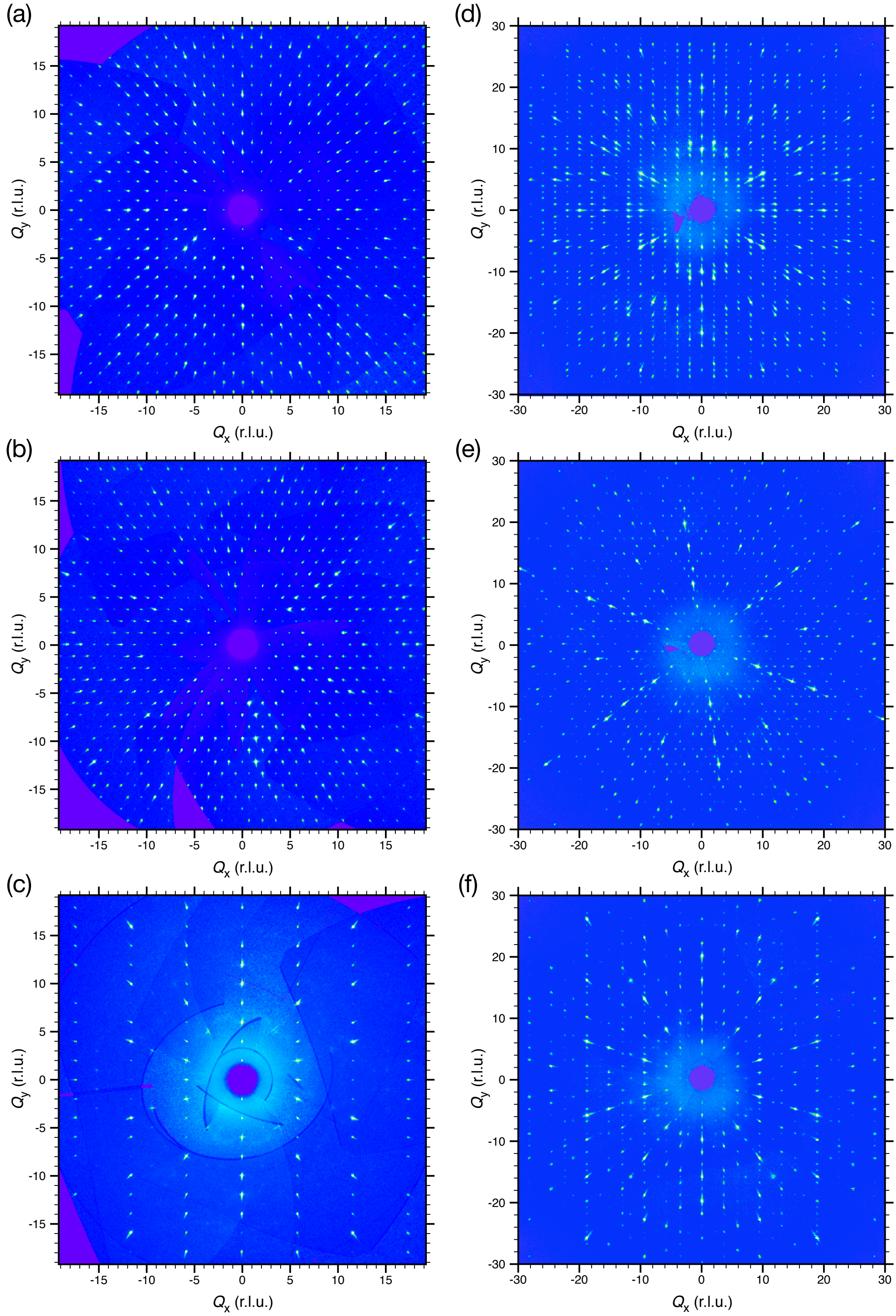

Figure S1; Reciprocal space sections perpendicular to (a) a twofold, (b) a threefold and (c) a pseudo-fivefold [3 5 0] directions for the Y-Cd-Mg 1/1 approximant, and (d) a twofold, (e) a threefold and (f) a pseudo-fivefold [5 8 0] directions for the Y-Cd-Mg 2/1.

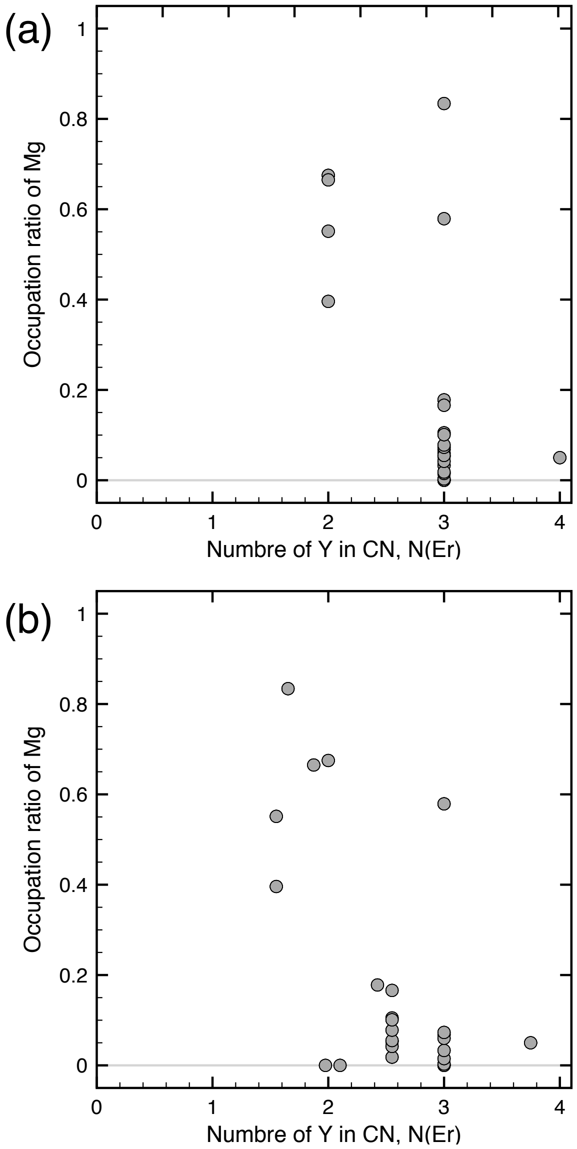

Figure S2; Correlations between Mg occupancy and local environment for M1–M26 in the Er-Cd-Mg 2/1 cAP. The occupation ratio of Mg is plotted against (a) CN(Er) and (b) N(Er).
